# Supplementary material for: Transcription Factor Activation Profiles (TFAP) identify compounds promoting differentiation of Acute Myeloid Leukemia cell lines
Source: Cell Death Discov. 2022 Jan 10;8:16. doi: 10.1038/s41420-021-00811-7 (PMC8748454; doi:10.1038/s41420-021-00811-7)
Supplement: Supplementary file 1 — Supplementary Material [file 41420_2021_811_MOESM1_ESM.docx]

**Supplementary Table 1:** Annotation of Haematopoietic transcription factors

| **TFs** | **Gene Name** | **Description** | **Reference (PMID)** |
| --- | --- | --- | --- |
| CCAAT/enhancer-binding protein alpha | *CEBPA* | Promotes granulocytic differentiation | 11242107 |
| E3 SUMO-protein ligase EGR2 | *EGR2* | Promotes myeloid leukemia cell differentiation | 1864967 |
| Early growth response protein 1 | *EGR1* | Promotes myeloid leukemia cell differentiation | 1864967 |
| Endothelial transcription factor GATA-2 | *GATA2* | Required for myeloid differentiation | 12433372 |
| Runt-related transcription factor 1 | *RUNX1* | Required for hematopoiesis | 17431401 |
| Transcription factor AP-1 | *JUN* | Required for myeloid differentiation | 8423806 |
| Transcription factor PU.1 | *SPI1* | Controls myeloid and lymphoid differentiation | 23868921 |

As previously described, t-SNE method is a non-linear dimensional reduction algorithm. We applied the tSNE algorithm on gene expression and transcriptional factor profiles of different cell lines treated with multiple compounds, in order to globally evaluate what is the biological feature that drives the samples.

In Supplementary Figure 1 each dot represents a sample. Samples that belong to the same cell line tend to be close together when the tSNE is applied on all gene expression profiles (1A), instead when we applied the tSNE on the transcription factor profiles (1B) we observe a major similarity between samples treated by the same compound and a reduction of the cell-line background influence.

The improved compound similarity in Figure 1B is demonstrated in the sample distance distribution analysis reported in Figure 2 (main text).

**
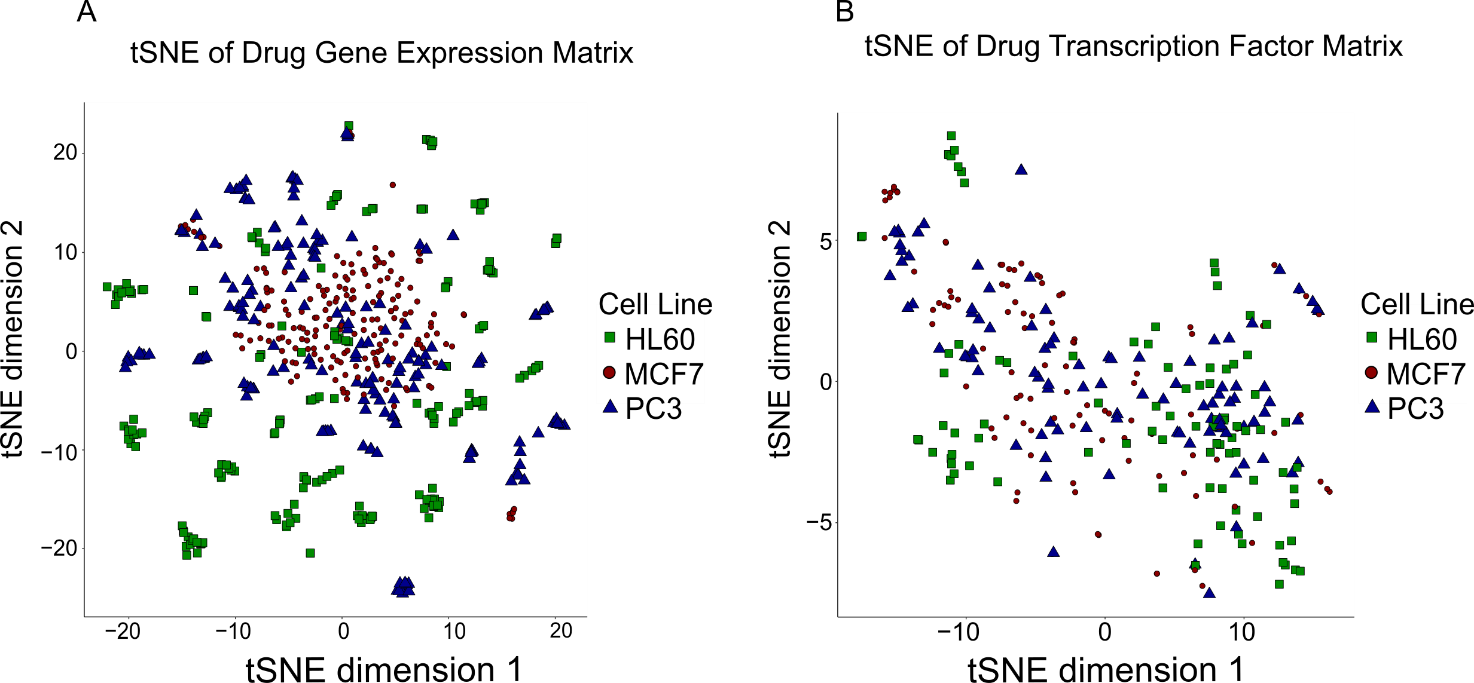
**

**Supplementary Figure 1**. tSNE maps of the multidimensional transcriptional profiles of different cell lines are incubated with different drugs. Each point correspond to different drug. Cell lines are represented with different shape and color. **(A)** tSNE map of gene expression profiles. **(B)** tSNE map of transcription factor profiles.

**
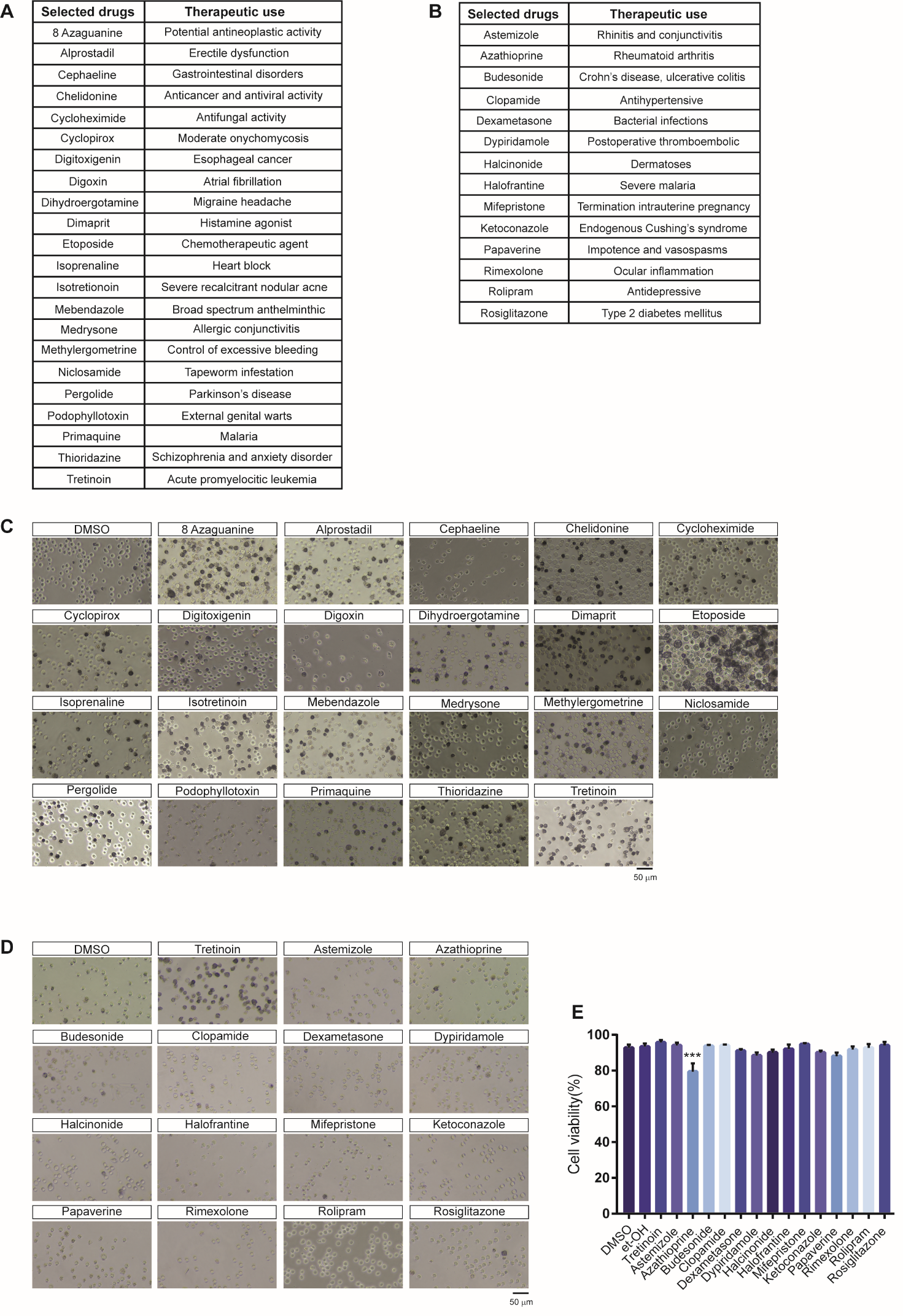
**

**Supplementary Figure2. (A)** List predicted drugs annotated with their therapeutic use. **(B)** List of the randomly chosen drugs that were tested for their ability to induce the differentiation of HL-60 cells. **(C)** Representative light microscope images of NBT staining in HL-60 after four days of the 22 selected drug treatment. Scale bar is 50 μm. **(D)** Representative light microscope images of NBT staining in HL-60 after four days of the randomly chosen drugs treatment. Scale bar is 50 μm. **(E)** Cell viability after four days of the randomly chosen drugs treatment. Cell viability was assessed by the Trypan blue exclusion test. Data are represented as means of three biological replicates ± SEM.Statistical analysis was performed using One-way ANOVA. Data are presented as mean ± SEM of three biological replicates. Significance * p ≤ 0.05, ** p ≤ 0.01, *** p ≤ 0.001, **** p ≤ 0.0001 and are related.
